# Motor coordination deficits in *Alpk1 *mutant mice with the inserted *piggyBac *transposon

**DOI:** 10.1186/1471-2202-12-1

**Published:** 2011-01-05

**Authors:** Muyun Chen, Rener Xu

**Affiliations:** 1 Institute of Developmental Biology & Molecular Medicine, School of Life Sciences, Fudan University, Shanghai 200433, China

## Abstract

**Background:**

ALPK1 (α-kinase 1) is a member of an unconventional alpha-kinase family, and its biological function remains largely unknown. Here we report the phenotypic characterization of one mutant line, in which the *piggyBac *(*PB*) transposon is inserted into the *Alpk1 *gene.

**Results:**

The *piggyBac*(*PB*) insertion site in mutants was mapped to the first intron of the *Alpk1 *gene, resulting in the effective disruption of the intact *Alpk1 *transcript expression. The transposon-inserted *Alpk1 *homozygous mutants (*Alpk1^PB/PB^*) displayed severe defects in motor coordination in a series of behavioral analysis, including dowel test, hanging wire test, rotarod analysis and footprint analysis. However, the cerebellar architecture, Purkinje cell morphology and electrophysiology of the Purkinje cells appeared normal in mutants. The motor coordination deficits in the *Alpk1^PB/PB ^*mice were rescued by transgenic mice expressing the full-length *Alpk1*-coding sequence under the control of the ubiquitous expression promoter.

**Conclusions:**

Our results indicate that ALPK1 plays an important role in the regulation of motor coordination. *Alpk1^PB/PB ^*mice would be a useful model to provide a clue to the better understanding of the cellular and molecular mechanisms of ALPK1 in the control of fine motor activities.

## Background

The alpha kinase has been initially identified and characterized in *Dictyostelium discoideum *as myosin heavy chain kinase [1-3]. Unlike many conventional protein kinases, which phosphorylate the protein sites embedded in beta-sheet secondary structures[4]; the phosphorylation sites in the substrates of the alpha kinase tended to be in the configuration of an alpha helix[5,6]. The alpha kinase was hypothesized to be the consequence of recent evolution, and believed to play key roles in complex signaling transductions in higher organisms [7,8]. At present, a total of six alpha kinase members have been identified in the vertebrates, including elongation factor-2 kinase (eEF2k), subfamily M member 6 and 7 of transient receptor potential cation channel (TRPM6 and TRPM7), and alpha-kinase 1-3 (ALPK1-3)[3,9]. Eukaryotic elongation factor-2 kinase (eEF2K) is a Ca^2+ ^and calmodulin-dependent kinase[10], regulating the global protein translation [11]. The activity of eEF2K was also reported to be modulated by the mTOR and AMPK signaling pathways [12-14]. TRPM6 and TRPM7 have similar protein structures, both consisting of transient receptor potential (TRP) cation channels in the N-terminal and alpha-kinase domain in the C-terminal[15]. TRPM6 is important for maintaining whole body Mg^2+ ^levels[16], while TRPM7 might be involved in the Ca^2+ ^signaling [17]. ALPK1, ALPK2 and ALPK3 all carry the alpha-kinase domains in the C-terminal[18]. ALPK1 was shown to phosphorylate the myosin IA and play a role in the apical vesicle transport in epithelial cells [19]. The functions of ALPK2 and ALPK3 are largely unknown.

*PiggyBac*, a DNA transposon, was originally found in cabbage looper moth *Trichoplusiani*[20,21] and reported recently as an useful genetic manipulation tool in mice[22]. In the present study, we characterized the *Alpk1 *insertedmice and found that in a series of behavioral analyses, severe motor coordination deficits were observed in the *Alpk1^PB/PB ^*mice, indicating that ALPK1 may play an important role in the control of the fine motor activity.

## Results

### Genetic characterization of *Alpk1^PB/PB ^*mutant mice

**Figure 1:**
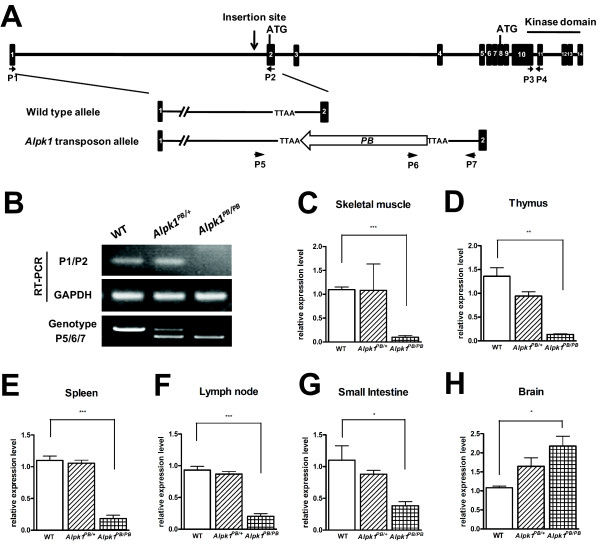
**Characterization of the *PB *insertion site in *Alpk1^PB/PB ^*mice**. (A) Position of the *PB *insertion. *PB *integration site in *Alpk1^PB/PB^*mice was mapped to the first intron of *Alpk1 *gene on mouse chromosome 3, nucleotide 128212040 (ENSMUSG00000028028, NCBI m37). Diagram shows the genomic structure of *Alpk1 *gene with introns (lines) and exons (black boxes). Two putative start codons and kinase domain are indicated above; gene specific primers are indicated below. TTAA: the four nucleotides required for *PB *insertion. (B) Disruption of endogenous *Alpk1 *transcript in *Alpk1^PB/PB ^*mice. *Alpk1 *5'-end transcript in wild type (WT), *Alpk1^PB/+ ^*and *Alpk1^PB/PB ^*mice were analyzed by RT-PCR using the indicated primers P1/P2. cDNAs from whole-brain were amplified as templates. GAPDH was used as an internal control. The different genotypes were determined by 3-primer PCR to detect WT (801 bp) and *PB *insertion alleles (631 bp). (C-H) Alteration of *Alpk1 *3'-end transcript levels in *Alpk1^PB/PB^*mice. The transcription level of *Alpk1 *3'-end transcript in skeletal muscle (C),thymus (D), spleen (E), lymph node (F), small intestine (G)and brain (H) were measured by real-time quantitative RT-PCR using the indicated primers P3/P4. *Alpk1*mRNA expression levels were normalized to GAPDH. *P < 0.05, ***P < 0.001, n = 3 per group.

One *PB *insertion line, H362cR1, was mapped and found that *PB *transposon was inserted into the first intron of the *Alpk1 *gene (Figure 1A). By using RT-PCR with primer pairs located on exon 1 and exon 2 of the *Alpk1 *gene to amplify the 5'-end transcript, the lack of the intact *Alpk1 *transcript was observed in homozygous mice (*Alpk1^PB/PB^*) (Figure 1B), indicating that the endogenous *Alpk1 *transcript was disrupted by the *PB *insertion. To examine the expression level of the *Alpk1 *coding sequence, real-time quantitative PCR (qPCR) was applied to quantify the 3'-end transcripts with primer pairs located on exon 10 and exon 11 of the *Alpk1 *gene. The transcription level of *Alpk1 *was decreased in many tissues, including skeletal muscle (Figure 1C), thymus, spleen, lymph node and small intestine (Figure 1D, E, F and 1G), whereas it was increased in the brain (Figure 1H).

**Figure 2:**
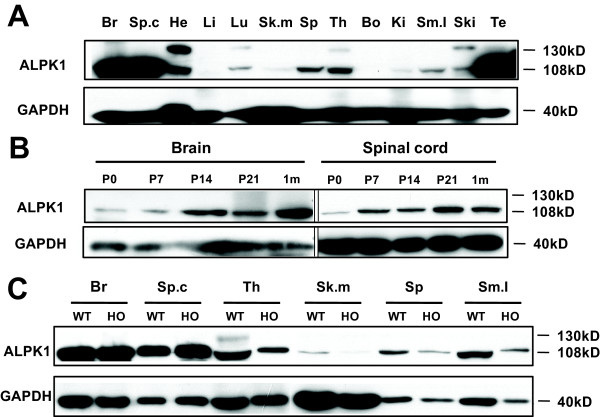
**Expression pattern of ALPK1 in wild type and *Alpk1^PB/PB^*mice**. (A) Immunoblots were performed on different tissues from adult wild type mice by using anti-ALPK1 antibody. ALPK1 short isoform (108 kD) was highly expressed in brain, spinal cord, heart, lung, spleen, thymus, small intestine, skin and testis, while detectable inskeletal muscle and kidney. ALPK1 large isoform (130 kD) appeared in heart, lung, thymus and skin.(B) Immunoblots were performed on brain and spinal cord extracts from different aged wild type mice. Only 108 kD isoform of ALPK1 was detected in mouse brain and spinal cord. P0, P7, P14 and P21 stand for postnatal day 0, 7, 14 and 21, respectively. 1 m: one month. (C) Immunoblots were performed on severaltissues from adultwild type (WT) and *Alpk1^PB/PB ^*mice (HO)by using anti-ALPK1 antibody. In skeletal muscle, spleen, small intestine and thymus, the expression level of ALPK1 was dramatically decreased in *Alpk1^PB/PB^*mice, while in brain and spinal cord, the expression level of ALPK1 were increased. **Br**, brain;**Sp.C**, spinal cord; **He**, heart; **Li**, liver; **Lu**, lung; **Sk.m**, skeletal muscle; **Sp**, spleen; **Th**, thymus; **Bo**, bone; **Ki**, kidney; **Sm.I**, small intestine; **Ski**, Skin; **Te**, testis; **M**, protein marker.

By using western blot with the rabbit polyclonal antibody specific to the C-terminal of ALPK1 (generated and purified in this study, described in Materials and Methods), we found that ALPK1 was expressed ubiquitously, consistent with the expression profiling in the mouse microarray analyses http://symatlas.gnf.org/SymAtlas/. Two protein isoforms were detected with molecular weight of about 130 kD and 108 kD, which are consistent with the protein sizes predicted by the mouse genome database (Figure 2A and 2B). The108 kD isoform was highly expressed in the brain, spinal cord, heart, lung, spleen, thymus, small intestine, skin and testis, while detectable in skeletal muscles and kidneys. The 130 kD isoform was found in the heart, lung, thymus and skin. Furthermore, the ALPK1 protein levels in *Alpk1^PB/PB ^*mice were decreased in most of the tissues except in the brain and the spinal cord, where the protein levels increased (Figure 2C and also see below), consistent with the results of qPCR.

### Impaired motor coordinationin *Alpk1^PB/PB^*mice

**Figure 3:**
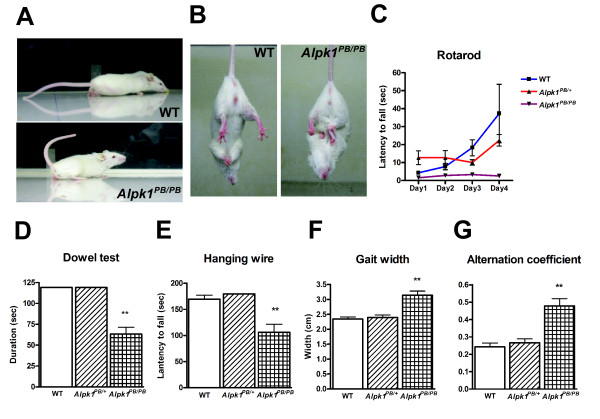
**Motor coordination deficits in *Alpk1^PB/PB ^*mice**. (A) Illustration of tail elevation in the walking *Alpk1^PB/PB ^*mice. (B) Illustration of feet-clasping behavior in *Alpk1^PB/PB ^*mice. The *Alpk1^PB/PB^*mice exhibited feet-clasping posture (right) compared to normal posture in wild type mice (left) when the tails were suspended. (C) Complete loss of motor coordination in *Alpk1^PB/PB ^*mice in the rotarod test. *Alpk1^PB/PB^*mice failed to stay on the rotating rod even after a four-day consecutive trial. (D-G) Impaired performances of *Alpk1^PB/PB^*mice in the dowel test (D), in the hanging wire test (E) and in the footprint test (F and G). *P < 0.05, **P < 0.01, ≥5 per group.

Compared to the wild type mice, the *Alpk1^PB/PB^*mutants exhibited elevated tail posture during walking, and occasionally their tails were bent forward (Figure 3A and see additional file 1 and 2). Feet-clasping was observed when the *Alpk1^PB/PB ^*mice were suspended by their tails (Figure 3B). A series of behavioral tests was performed to examine motor control in mutants. The *Alpk1^PB/PB^*mice could not stand on the rotating rod on the rotarod test (Figure 3C). In the dowel test, the *Alpk1^PB/PB ^*mice could not easily keep balance on the fixed wooden bar (Figure 3D). The retention time of the *Alpk1^PB/PB ^*micein the hanging wire test was much less than those of the *Alpk1^PB/+ ^*or wild type mice(WT: 169.7 ± 7.5 s, *Alpk1^PB/+^*: 179.8 ± 0.2s, *Alpk1^PB/PB^*: 106 ± 15.6 s)(Figure 3E). In the footprint test, the step width of the *Alpk1^PB/PB ^*mice was wider than that of the control mice (WT: 2.34 ± 0.07 cm, *Alpk1^PB/+^*: 2.40 ± 0.08 cm, *Alpk1^PB/PB^*: 2.93 ± 0.23 cm) (Figure 3F), and the alternation coefficient index also showed significant differences between the *Alpk1^PB/PB ^*mice and the controls (WT: 0.24 ± 0.02, *Alpk1^PB/+^*: 0.27 ± 0.02, *Alpk1^PB/PB^*: 0.48 ± 0.04) (Figure 3G). All these results implied that the *Alpk1 *gene may play an important role in motor coordination.

Mating of the *Alpk1^PB/+ ^*mice yielded a near-Mendelian distribution of genotypes in the offspring, and gender ratio of offspring was normal. The survival proportion of the *Alpk1^PB/PB ^*mice was similar to that of wild type controls until they were one year old.

### Cerebellar morphology and function in *Alpk1^PB/PB ^*mice

**Figure 4:**
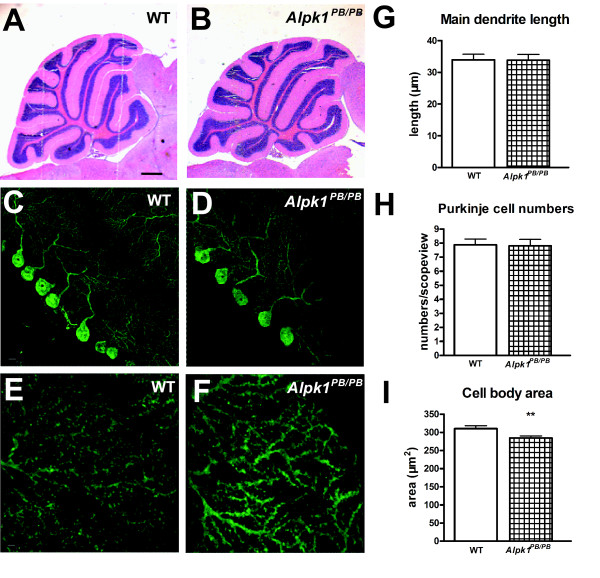
**Cerebellar architecture and morphology of Purkinje cells in *Alpk1^PB/PB ^*mice**. (A, B) H&E staining of the cerebellar cross sections from 6-week-old *Alpk1^PB/PB ^*mice and their wild type littermates. No gross abnormalities were found in the *Alpk1^PB/PB ^*mice. Scale bar: 500 μm. (C-F) Confocal images showing Purkinje cell morphology identified by anti-Calbindin immunofluorescence staining of the cerebellar slices from 6-week-old *Alpk1^PB/PB ^*mice and their wild type littermates. Purkinje cell alignment and dendrite outgrowth (C, D) and Purkinje cell dendritic distal branches (E, F) displayed no obvious differencesbetween the *Alpk1^PB/PB ^*mice and their wild type littermates. All images are representatives of five sections from at least 3 mice per genotype. Scale bar, C, D: 10 μm; E, F: 2 μm. (G-I) Statistic analysis of Purkinje cell morphology. High-resolution confocal image stacks of cerebellar slices from 6-week-old *Alpk1^PB/PB ^*mice and their wild type littermates were processed by Image Pro Plus analysis. Main dendrite length (G), visible Purkinjce cells per scopeview (H) and area of Purkinje cell body (I) were quantified. Data are mean ± SEM of about 200 cells from several cerebellar slices of at least two individual mice per genotype. Experimenter was blind to the genotypes. **P < 0.01.

Since the cerebellum plays an important role in the coordination of movements[23], multiple aspects of the cerebellum were examined in *Alpk1^PB/PB ^*mice, including the cerebellar architecture, Purkinje cell morphology and the electrophysiological properties of the Purkinje cell. Foliation and lamination of the cerebellar cortex appeared normal in *Alpk1^PB/PB^*mice by histological analysis on cerebellar sections (Figures 4A and 4B). Immunofluorescence staining with anti-calbindin antibody was performed to examine Purkinje cell morphology in details. The alignment of Purkinje cells between the granular and molecular layers (Figures 4C and 4D), Purkinje cell dendritic branches (Figures 4E and 4F), dendritic length (Figure 4G) andPurkinje cell numbers (Figure 4H) appeared normal in *Alpk1^PB/PB ^*mice compared tothe wildtype controls. Purkinje cell body in *Alpk1^PB/PB ^*mice was smaller than those of the controls (Figure 4I). The cerebellar long-term depression (LTD) did not show significant differences between the *Alpk1^PB/PB ^*mice and the wild type controls (see additional file 3).

### Transgenic rescue of defective motor coordination in *Alpk1^PB/PB ^*mice

**Figure 5:**
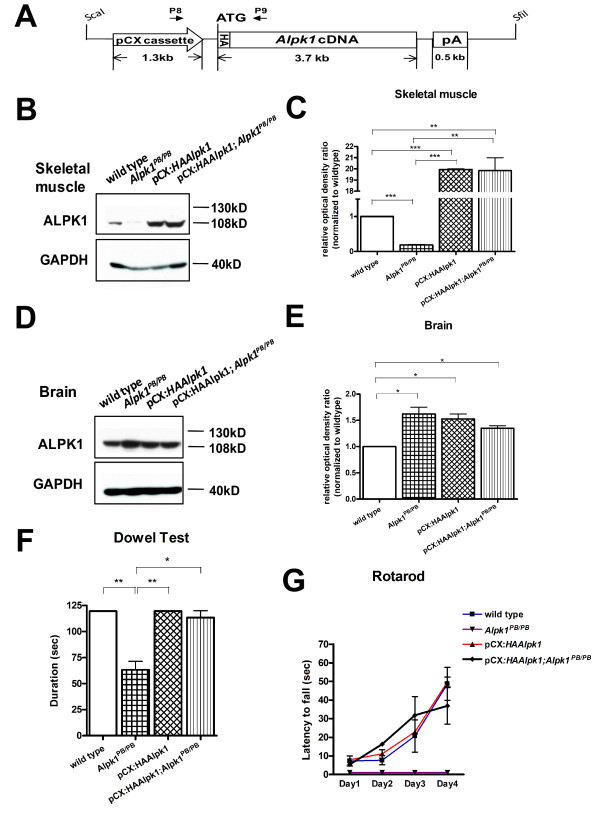
**Transgenic rescue of motor coordination deficits in *Alpk1^PB/PB^*mice**. (A) Diagram of *Alpk1 *transgene construct. The full-length coding sequence of *Alpk1*, fused with HA tag, was introduced into the expression vector containing pCX cassette (consisting of a chicken beta-actin promoter and a 5' truncated exon and intron of beta-actin) and a 3' rabbit beta-globin polyadenylation signal. Indicated restriction enzymes ScaI and SfiI were used to excise the transgene from the construct for micro-injection. The transgene-specific PCR primers P8/P9 were used for the transgenic genotyping. (B-E) Analysis of ALPK1 expression in mouse tissues from compound genotypes. Representative western blots showing total ALPK1 proteins in skeletal muscle (B) and brain (D) derived from the indicated genotypes by using anti-ALPK1 antibody. In panel (C, muscle) and panel (E, brain), histogram values represent the ALPK1 densitometric immunoreactive intensity normalized to the intensity of the wild type tissue sample on the same blot.*P < 0.05, ***P < 0.001, n = 3 for each group. (F and G) Behavioral performance of mice with compound genotypes in the dowel test (F) and the rotarod test (G). The motor coordination deficits were completely rescued in the pCX:*HAAlpk1*;*Alpk1^PB/PB ^*mice.*P < 0.05, ***P < 0.001, ≥5 per group.

Hemagglutinin (HA)-tagged murine full-length *Alpk1 *coding sequence driven by the ubiquitously expressed chicken beta-actin promoter cassette (pCX) was constructed to generate transgenic mice (Figure 5A). As the HA tag was fused to the amino-terminal end of ALPK1, only the 130 kD protein isoform of transgene could be directly detected by using anti-HA antibody in western blot. Indeed, the 130 kD isoform of transgene expression was detected in the thymus by anti-HA immunoblotting (see additional file 4). In some tissues, such as the skeletal muscle and brain, ALPK1 only expressed in 108 kD short isoform, excluding the possibility for detection of transgene expression by anti-HA immunoblotting. Therefore, comparison of densitometric immunoreactive intensity in the anti-ALPK1 immunoblots was utilized to verify transgene expression in those tissues. The relative prevalence of total ALPK1 immunoreactivity in skeletal muscle from the *Alpk1^PB/PB ^*mice was 0.18 ± 0.01 times than that of wild type controls. In comparison, the levels of ALPK1 expression in skeletal muscle from the pCX:*HAAlpk1 *and the pCX:*HAAlpk1*;*Alpk1^PB/PB ^*mice were 19.95 ± 0.05 and 19.85 ± 1.15 times than wild type controls (Figure 5B and 5C), indicating that the transgene was highly expressed in skeletal muscle. In the brain, the relative prevalence of total ALPK1 immunoreactivity from pCX:*HAAlpk1 *mice was 1.52 ± 0.09 times than that of wild type controls, suggesting that the transgene was expressed in the brain. The levels of ALPK1 expression in brain from the *Alpk1^PB/PB ^*and the pCX:*HAAlpk1*;*Alpk1^PB/PB ^*mice were 1.62 ± 0.13 and 1.36 ± 0.05 times than that of wild type controls, respectively (Figures 5D and 5E).

In the behavioural tests, the performance of pCX:*HAAlpk1*;*Alpk1^PB/PB ^*was similar to wild type controls in the dowel test (Figure 5F) and in the rotarod test(Figure 5G), indicating that the transgenic ALPK1 could rescue motor coordination deficits in *Alpk1^PB/PB ^*mice.

## Discussion

ALPK1, also known as lymphocyte alpha-kinase, was initially identified in the human lymphocyte cDNA library[3]. Our anti-ALPK1 immunoblot results confirmed that ALPK1 was highly expressed in lymphoid organs, such asthymus and spleen, implicating that ALPK1 might function in the development of the immune system. Moreover, the expression level of ALPK1 in lymphoid organs was significantly decreased by *PB *insertion in *Alpk1^PB/PB ^*mice, leading to speculation as to whether the immune system may be affected in mutants. FACS analysis of different markers on CD4^+^, CD8^+ ^and B cellswere performed, and the proportion of T and B lymphocyte populationsin *Alpk1^PB/PB ^*mice was not changed compared to those of the wild type controls (data not shown). Further studies may be required to assess whether ALPK1 plays a role in the immune system.

Besides motor coordination deficits, the *Alpk1^PB/PB ^*mice also have other interesting abnormalities. The *Alpk1^PB/PB ^*mice exhibited mild thoracolumbar kyphosisby micro-CT scanning (data not shown). However, further analysis on bone density, bone trabecula, and the structure of sacroiliac joint presented no differences between the *Alpk1^PB/PB ^*and the wild type mice (data not shown), implying that the kyphosis may be attributed to other causes apart from bone development. The *Alpk1^PB/PB ^*mice also showed male infertility. Histological analysis revealed that the testes in the *Alpk1^PB/PB ^*mice seemed to develop normally. Sperm derived from *Alpk1^PB/PB^*or wild type mice was used for *in vitro *fertilization, and no significant differences in the efficacy of offspring production were observed between the two groups (data not shown). When videotaping the sexual behavior in mice (according to the protocol described [24]), we found that the male *Alpk1^PB/PB ^*mice could not properly mount the female mice, leading to afailure of the mating process. Abnormal mounting ability in the male *Alpk1^PB/PB ^*mice is likely associated with motor coordination deficits in the mutants.

ALPK1 protein levels were increased in the brain of *Alpk1^PB/PB ^*mice. However, several lines of evidence went against the hypothesis that motor coordination deficits in the *Alpk1^PB/PB ^*mice may be caused by the increased protein levels in mutant brains. First, our results showed that two protein isoforms of ALPK1 (130 kD and 108 kD) presented in a tissue-dependent manner, while only 108 kD isoform could be detected in brains from both *Alpk1^PB/PB^*and the wild type mice. In order to distinguish the slight differences of ALPK1 proteins in the *Alpk1^PB/PB^*and wild type mice brains, 2D-PAGE analysis was performed. One spot with the same migration position could be detected in the gel by our anti-ALPK1 antibody (data not shown), indicating that the elevated 108 kD isoforms in brains of the *Alpk1^PB/PB^*mice may be biochemically similar or identical to those produced by the wild type control brains, and *PB *insertion in the *Alpk1^PB/PB^*mice might not affect the translation initiation site of *Alpk1*transcript in the brain. Second, similar to the situation seen in the *Alpk1^PB/PB^*mice, only the 108 kD isoforms could be detected in the brain extracts from transgenic mice. Nevertheless, the transgenic line alone did not exhibit the defective motor coordination. Third, multiple aspects of the cerebellumwhich have beenproved to play a key role in motor coordinationwere examined in the *Alpk1^PB/PB^*mice. No significant differences were observed between the mutants and the wild type controls, implicating that thedefective motor control in mutants may act in a cerebellum-independent manner. The change of the expression of ALPK1 in skeletal muscle was consistent with the appearance/disappearance of the motor coordination deficits in the mice with different genotypes. However, no obvious differences were observedin the histological analysis of the skeletal musclebetween the wild type and *Alpk1^PB/PB^*mice (data not shown). At present, the mechanisms underlying the severe motor coordination deficits in the *Alpk1^PB/PB^*mice remain elusive. Tissue-specific transgenic mice would be necessary for further investigations to narrow down the affected tissues in the *Alpk1^PB/PB^*mice.

## Conclusions

In the present study, mice for *Alpk1^PB/PB ^*alleles were phenotypically characterized and severe motor coordination defects were observed in the *Alpk1^PB/PB ^*mice in multiple behavioral tests. Transgenic mice expressing full-length murine coding sequence of ALPK1 were capable of rescuing the motor deficits in mutants. No obvious differences in cerebellar architecture, fine structure and LTD of Purkinje cells were found in the *Alpk1^PB/PB^*mice. In summary, our *Alpk1 *inserted mice provided the first genetic evidence that ALPK1 may play a crucial role in motor coordination. The *Alpk1^PB/PB ^*mice provided a valuable tool to elucidate the mechanisms of ALPK1 in the regulation of motor coordination.

## Methods

### Mouse procedure

The mice used in this paper are on the FVB/NJ background and were maintained on a 12/12 h light/dark cycle with food and water available *ad libitum*. The mouse handling were reviewed and approved by the Animal Care and Use Committee of the Institute of Developmental Biology and Molecular Medicine at Fudan University. The *piggyBac *inserted ALPK1 mouse line was kindly provided by Dr. TianXu and Xiaohui Wu from our institute. The *PB*transposon was inserted in the first intron of Alpk1 on mouse Choromsome 3, nucleotide 128212040, and the direction of the insertion was opposite to the gene location.

### Mapping *PB *inserted Alpk1 allele

Offspring with the transposon inserted into the *Alpk1 *gene were identified by 3-primers genotyping PCR using the primers P5/P6/P7 as indicated. The primer sequences were upon request. PCR conditions were as follows: initial denaturation at 93°C for 90 sec; 40 cycles of 93°C for 30 sec, 57°C for 30 sec, 65°C for 3 min; and a final extension at 65°C for 10 min. This condition was used for all the PCRs described, except where otherwise noted. Genomic DNA extracted from mouse toes were used as templates.

### RT-PCR and Real-time quantitative PCR

Mouse tissues were harvested and total RNA was extracted using TRIzol (Invitrogen) and treated with RNase-free DNaseI (TaKaRa) to eliminate genomic DNA contamination. cDNA was synthesized from total RNA (400 ng) by using AWV RNA PCR Kit (TaKaRa) following manufacturer's protocols. To examine the disruption of inserted *Alpk1 *mRNA, cDNA was amplified using the primers P1/P2 located within exon 1 and exon 2. GAPDH was used as an internal control.

To quantify the *Alpk1 *expression levels in different tissues, the PCR amplifications of different cDNAs by using primers P3/P4 were performed with 2X HotSybr PCR Reaction Mix (NuStar Laboratory) on the Mx3000P Quantitative PCR System (Stratagene) following the manufacturer's instructions, SYBR green used as fluorescent dye. The amplification conditions were as follows: initial incubation at 95°C for 15 min, followed by 40 cycles of denaturation at 94°C for 15 sec, annealing for 30 sec, and extension at 72°C for 30 sec. Melting curve analysis was then performed to verify the specificity of the PCR products. The quantification of target mRNA was achieved in triplicate according to the standard curve method with GAPDH as a calibrator.

### Generation of anti-ALPK1 antibody

The DNA fragment coding for the ALPK1 region (amino acid 801-918) was PCR amplified from the *Alpk1*cDNA, and then cloned into pET32a (Novagen) for standard protein expression and purification. Polyclonal antibodies were raised by immunizing rabbits with the purified fusion proteins and affinity-purified with Hitrap NHS-activated HP columns (Amersham Biosciences).

### Generation of pCX:*HAAlpk1 *transgenic mice

The HA-tagged murinefull-length *Alpk1*coding sequence (RT-PCR product of *Alpk1 *transcripts based on the information available at ENSMUST00000029662) was inserted into a pCX transgene shuttle vector [25]. This transgene construct waslinearized by ScaI and SfiI, resolved by agarose gel, purified and microinjected intopronuclei of fertilized eggs derived from FVB/NJ mice following standard protocols. Transgenic founders were identifiedby PCR with the transgene-specific primers P8/P9. A total of 15 transgene-positive founder mice were obtained and two of them with higher transgene expression level were selected to establish two individual transgenic lines. Each line was outcrossed with *Alpk1^PB/PB ^*to obtain mice with compound genotypes for further investigations.

### ALPK1 protein analysis

Protein extraction was prepared with the RIPA lysis buffer (Santa Cruz) according to manufacturer's instruction and quantified with the BCA™Protein Assay Kit (Pierce). Equal amounts of samples were separated by SDS/PAGE, transferred onto PVDF membranes (Millipore), and immunoblotted following standard protocols. ALPK1 expression in tissues was detected by chemiluminescence by using anti-ALPK1 antibody (generated in this study; 1:500) as the primary antibody, and HRP-conjugated goat anti-rabbit IgG (Santa Cruz; 1:4,000) as the secondary antibody. Comparable levels of loaded protein were reconfirmed by probing membranes with a GAPDH antibody (KangCheng Biotech; 1:10,000). Quantitative analysis was carried out with NIH ImageJ software.

### Immunocytochemistry and imaging

Mice were anesthetized and killed by transcardial perfusion with PBS followed by 4% paraformaldehyde in PBS. The cerebellums were removed, postfixed in 4%PFA in PBS and cryoprotected by immersion in 30% sucrose in PBS at 4°C. 20-μm sections were prepared by using a cryostat and stored briefly in PBS at 4°C. Sections were incubated in a blocking buffer (PBS with 10% goat serum,0.05% Triton X-100) for one hour, then incubated with mouse Calbindin-D 28 K antibody (Sigma) in a blocking buffer at 4°C overnight. After washing in PBS, sections were incubated with goat anti-mouseFITC (Chemicon) and DAPI in blocking buffer for 4 hr. at room temperature, washed 3 times in PBS, then mounted and analyzed by confocal microscopy. High-resolution confocal images of FITC-labeled Purkinje cells were taken with Leica TCS SP2 with a 63x/1.4NA oil immersion lens.Quantitative measurements were obtained from confocal image stacks by using Image Pro Plus software asdescribed in the previous study [26].

### Behavioral assays

In all experiments, only male mice were used. Meanwhile, the experimenters were blind to genotypes for all assays. When the mice were one month old, a series of behavioral analysis was conducted by using the battery of tests described below.

#### Dowel test

In the 2-minute interval, mice were put in the center of a 0.9-cm wide horizontal wooden dowel, the duration time on the dowel were calculated. If mice walked across and off of the dowel, they were placed back again onto the dowel.

#### Hanging wire test

In the 3-minute interval, mice were put on the screen while the wire bars are upside. Gently waving the screen in the air and letting the wire face the ground,forcing the mice grip the wires. The latency for the mice to fall down was calculated. Mice that fell in <10 sec were provided a second trial.

#### Rotarod test

We measured thetime that the mice were ableto remain on a longitudinally rotatingrod (10 revolutions per min, TianhuanInstruments). Mice experienced six3-min training cycles at the age of approximately1 month. Trained micethen received four trials at definedages to get an average score. A maximumcutoff time of 180 sec was set foreach trial.

#### Footprint test

Gait analysis was carried out on footprints, which were obtained by paintingthe hind feet of mice with nontoxic black paint and having them walkon paper along a 50-cm-long, 9-cm-wide runway, with 16-cm-highwalls on either side. Seven consecutive steps were recorded in terms of step length, step width, alternation coefficient and linear movement according to the protocol described[27].

### Statistical analysis

Data were comparedbyunpaired two-tailed Student's *t*-test, and shown as the mean ± SEM. The significance was set at p < 0.05. All statistical analyses and scientific graphing were conducted by using Graphpad Prism 4 software.

## Authors' contributions

RX led this project and wrote the manuscript. MC also led this project, performed all the experiments except the electrophysiology test and wrote the manuscript. All authors read and approved the manuscript.

## Supplementary Material

Additional file 1 **Movie of wild type mice walking in the test arena.avi**. The wild type mice dragged its tail at horizontal level during walking. Click here for file

Additional file 2 **Movie of *Alpk1^PB/PB^*mice walking in the test arena.avi**. Compared to the wild type mice, the *Alpk1^PB/PB^*mutants exhibited elevated tail posture during walking. Click here for file

Additional file 3 **LTD in wild type and *Alpk1^PB/PB^*mice cerebellar slices.png**. (A, B) Time course of EPSC amplitudes of Purkinje cells in slices from the wild type (WT) mice (A) and *Alpk1^PB/PB^*(B). The EPSC was evoked by stimulating PFs. WT, n = 5; *Alpk1^PB/PB^*, n = 6. (C) PF-PC LTD analysis between the wild type and *Alpk1^PB/PB^*mice. No significant difference was found between the two groups. Click here for file

Additional file 4 **Detection of expressed *Alpk1 *transgene in mouse thymus.png**. Western blotting analysis of the protein extracts from mouse thymus with different genotypes (as indicated). The upper panel showed ALPK1 immunoreactivity, while the lower panel showed HA immunoreactivity in samples from the same mouse. GAPDH was used as internal control. Click here for file
