# Supplementary figures and images for: Motor coordination deficits in Alpk1 mutant mice with the inserted piggyBac transposon
Source: BMC Neurosci. 2011 Jan 5;12:1. doi: 10.1186/1471-2202-12-1 (PMC3030534; doi:10.1186/1471-2202-12-1)

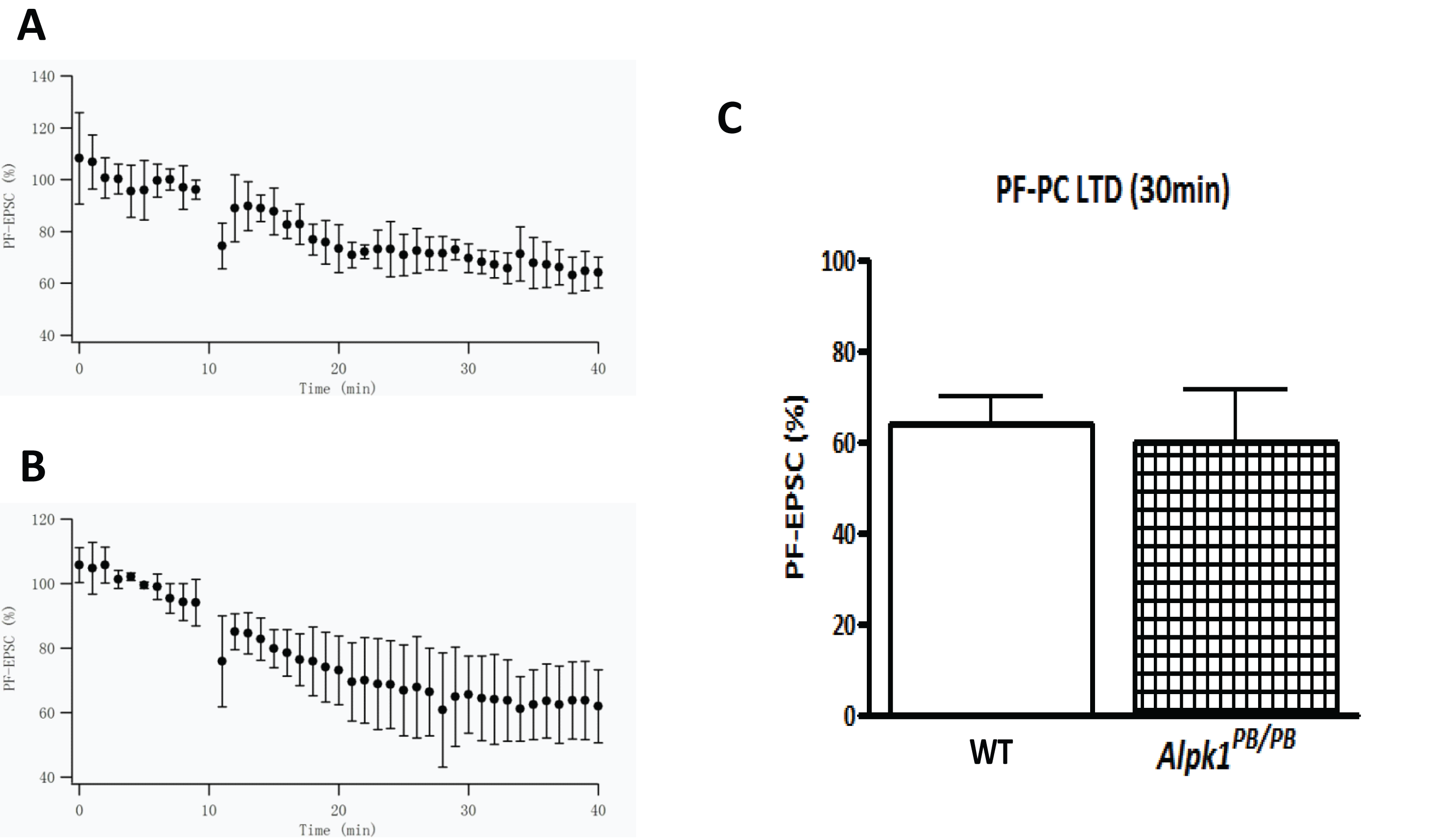

Supplement: Additional file 3 — LTD in wild type and Alpk1PB/PBmice cerebellar slices.png. (A, B) Time course of EPSC amplitudes of Purkinje cells in slices from the wild type (WT) mice (A) and Alpk1PB/PB(B). The EPSC was evoked by stimulating PFs. WT, n = 5; Alpk1PB/PB, n = 6. (C) PF-PC LTD analysis between the wild type and Alpk1PB/PBmice. No significant difference was found between the two groups. [file 1471-2202-12-1-S3.PNG]

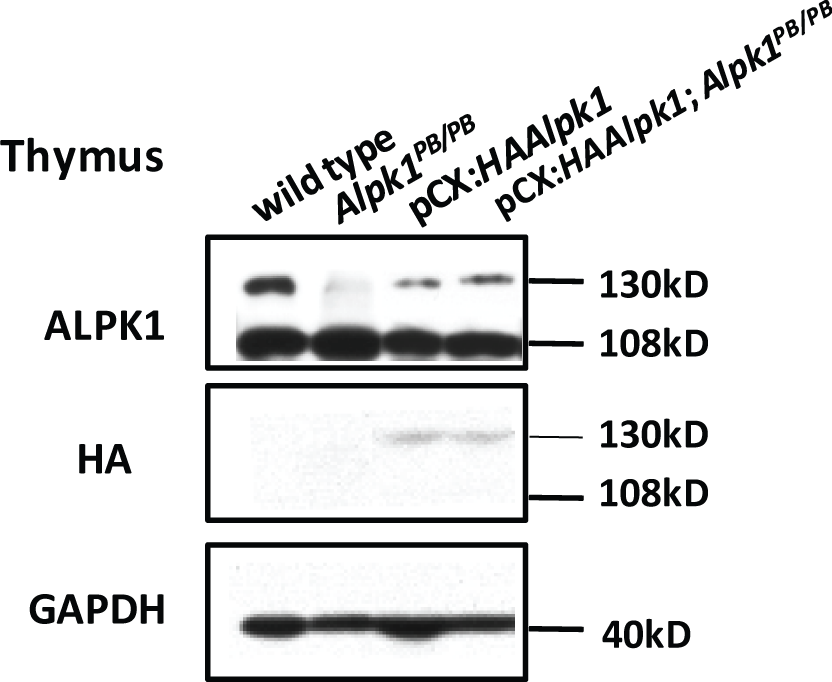

Supplement: Additional file 4 — Detection of expressed Alpk1 transgene in mouse thymus.png. Western blotting analysis of the protein extracts from mouse thymus with different genotypes (as indicated). The upper panel showed ALPK1 immunoreactivity, while the lower panel showed HA immunoreactivity in samples from the same mouse. GAPDH was used as internal control. [file 1471-2202-12-1-S4.PNG]
